# Some Liked It Hot: A Hypothesis Regarding Establishment of the Proto-Mitochondrial Endosymbiont During Eukaryogenesis

**DOI:** 10.1007/s00239-017-9809-5

**Published:** 2017-09-15

**Authors:** Cory D. Dunn

**Affiliations:** 1 Institute of Biotechnology, Helsinki Institute of Life Science, University of Helsinki, P.O. Box 56, 00014 Helsinki, Finland; 2 College of Sciences, Koç University, 34450 Sarıyer, İstanbul Turkey

**Keywords:** Endosymbiosis, Eukaryogenesis, Mitochondria, Archaea, Temperature, Bioenergetics

## Abstract

Eukaryotic cells are characterized by a considerable increase in subcellular compartmentalization when compared to prokaryotes. Most evidence suggests that the earliest eukaryotes consisted of mitochondria derived from an α-proteobacterial ancestor enclosed within an archaeal host cell. However, what benefits the archaeal host and the proto-mitochondrial endosymbiont might have obtained at the beginning of this endosymbiotic relationship remains unclear. In this work, I argue that heat generated by the proto-mitochondrion initially permitted an archaeon living at high temperatures to colonize a cooler environment, thereby removing apparent limitations on cellular complexity. Furthermore, heat generation by the endosymbiont would have provided phenotypic flexibility not available through fixed alleles selected for fitness at specific temperatures. Finally, a role for heat production by the proto-mitochondrion bridges a conceptual gap between initial endosymbiont entry to the archaeal host and a later role for mitochondrial ATP production in permitting increased cellular complexity.

## Introduction

Available evidence suggests that two prokaryotes, an archaeon and a bacterium, collaborated (Margulis 1970; Stanier 1970; Schwartz and Dayhoff 1978; Doolittle 1980; McInerney et al. 2014) in the eventual formation of nucleated cells with arguably (Booth and Doolittle 2015) increased complexity of form and function. However, the mechanisms leading to formation of eukaryotes remain a mystery (Koonin 2015; López-García et al. 2017; Zachar and Szathmáry 2017; Martin et al. 2017).

Mitochondria are eukaryotic organelles derived from α-proteobacterial endosymbionts capable of generating ATP by oxidative phosphorylation (Gray 2012). The earliest eukaryote likely harbored mitochondria, since all characterized eukaryotic lineages show evidence of containing (van der Giezen 2009), or having once contained (Karnkowska et al. 2016), these organelles. Consequently, it has been argued that mitochondria, and particularly the ATP that can be generated by these compartments, permitted an expanded number of proteins, an augmented phagocytic capacity, an increase in overt specialization, and the eventual formation of complex multicellular organisms (Lane and Martin 2010; Lane 2017; Martin et al. 2017). However, the relationship between mitochondrial ATP generation and genome expansion has been a matter of contention (Lynch and Marinov 2015, 2017). Moreover, how and why an endosymbiont not yet converted to an organelle might purposefully provide ATP to its host is not clear (Martin and Müller 1998).

Here, I propose that the initial driving force allowing maintenance of the proto-mitochondrial endosymbiont within its archaeal host was production of heat, thereby permitting endurance of lower temperatures. Only afterward did ATP generation by the early mitochondrion contribute to the increased apparent complexity exhibited by eukaryotes.

## Ancestral Archaea are Hyperthermophilic

High temperatures likely hastened the formation of ancestral life by accelerating reactions required for metabolism prior to the evolution of more specific and efficient enzymes (Wolfenden 2014). Today, while eukaryotes are not found at temperatures higher than ~60 °C (Brock 1967; Forterre 2013), prokaryotic cells can proliferate at temperatures even exceeding 120 °C (Takai et al. 2008). Although some bacteria are hyperthermophiles, most enumerated hyperthermophilic prokaryotes that proliferate above 80 °C are archaea, and the ancestral state of archaea is almost certainly hyperthermophily (López-García et al. 2015; Akanuma 2017). Only later were archaea able to populate environments of lower temperature, including habitats close to the freezing point of water (Cavicchioli 2006).

As archaea moved to lower temperatures, they were likely to encounter multiple challenges presented by their new environment. Most prominently, cells residing at lower temperatures require enzymes with greater catalytic power than those selected at higher temperatures (Wolfenden 2014). How structural changes to enzymes promote greater catalysis at lower temperatures remains under investigation, but may include increased conformational flexibility or changes to thermodynamic factors associated with transition state formation (Sterner and Liebl 2001; Wolfenden 2014; Nguyen et al. 2017). In addition to new demands on the activity of fully folded enzymes, optimal pathways toward protein folding and assembly differ at lower temperatures, requiring compensation by mutation or by chaperone activity (Sterner and Liebl 2001).

Besides the considerable obstacles to protein function brought about by movement from higher to lower temperature, other consequences of a cooler setting are also apparent. For example, DNA at high temperature is prone to unwind, and in fact many hyperthermophiles express a reverse gyrase in an attempt to positively supercoil DNA (López-García et al. 2015). Any approach to maintaining high helical tension would be maladaptive as cells move to lower temperature. Changes in RNA dynamics are also likely to be consequential as hyperthermophiles reach lower temperatures, and at least one hyperthermophilic archaeon, *Thermococcus kodakaraensis*, harbors a cold-inducible RNA helicase (Shimada et al. 2009). In addition, the same archaeon has been demonstrated to alter its lipid content upon reduction of culture temperature by 30 °C (Matsuno et al. 2009), illustrating the necessity for prokaryotes to compensate for membrane fluidity differences at lower temperatures (Siliakus et al. 2017). Gas solubility and the stability of metabolites also scale with temperature (D’Amico et al. 2006; Wolfenden 2014), prompting a further need for adaptation. Notably, there may be a trend toward larger genomes as the optimal proliferation temperature of archaeal species decreases (Laksanalamai et al. 2004; Sabath et al. 2013), and a comprehensive analysis suggests that the protein evolution rate of archaea living at lower temperatures is elevated in comparison to hyperthermophilic archaea (Groussin and Gouy 2011). Taken together, these findings suggest many challenges for hyperthermophilic organisms potentially colonizing or traversing lower temperature environments, though leaving a high-temperature niche behind may remove barriers to genome expansion, variation, and phenotypic diversity.

One mechanism by which archaea appear to have adapted to reduced temperature is through abundant lateral gene transfer (LGT) from mesophilic bacteria already residing at lower temperatures (López-García et al. 2015). Such gene transfers presumably promoted improved protein folding or enzyme activity as organisms moved to colder locations. For example, many ancestral hyperthermophilic archaea lack specific chaperones, such as Hsp70 proteins, that were later acquired during relocation to a lower temperature environment (Laksanalamai et al. 2004; Petitjean et al. 2012), suggesting that such chaperones may have initially promoted polypeptide folding or stability (López-García et al. 2004). Moreover, transfer of chaperone genes from a bacterium residing at low temperature, *Oleispira antarctica*, can promote proliferation of the more thermophilic *Escherichia coli* under cooler conditions (Ferrer et al. 2003). Beyond the assistance provided by LGT in improving proteostasis, metabolic enzymes selected to perform within hyperthermophiles may not retain sufficient catalytic activity at reduced temperature (Sterner and Liebl 2001; Nguyen et al. 2017), prompting the need for orthologous replacement by genes from other organisms.

Hyperthermophilic archaea were clearly able to establish themselves within lower temperature environments (Cavicchioli 2006; López-García et al. 2015), and also commonly transit colder climes in order to seed new locations at their preferred temperature (Wirth 2017). However, should the piecemeal lateral transfer or slow alteration of genetic information be the only path toward the endurance of reduced temperature? What if an archaeal cell could efficiently generate its own heat, allowing the maintenance of elevated intracellular temperature even when encountering colder habitats?

## Mitochondria Generate Heat

In prokaryotes and prokaryote-derived organelles, a proteinaceous electron transport chain (ETC) converts electronic energy to a proton gradient used to power mechanochemistry and to drive metabolite movement across membranes (West 1974; Junge and Nelson 2015; Nishihara and Kitao 2015). During operation of the ETC, some energy is inevitably dissipated as heat in the course of each electron transfer (Murphy 1989). Moreover, once protons are pumped across the mitochondrial inner membrane (IM) by the ETC, they can leak back across the IM in a heat-producing futile cycle (Brand 2000). Indeed, approximately a quarter of protons pumped by the ETC in several mammalian tissues are not coupled to performance of useful work, and the magnitude of proton leak can range to even higher levels in some tissues. While there is debate regarding the reliability of subcellular temperature measurements (Baffou et al. 2014, 2015; Kiyonaka et al. 2015; Suzuki et al. 2015), studies reliant upon divergent approaches to investigating subcellular temperature suggest that differences in temperature between mitochondria and the cytosol can be quite substantial (Okabe et al. 2012; Sakaguchi et al. 2015; Chretien et al. 2017; Nakano et al. 2017). Indeed, fully functional mitochondria in cultured human cells appear to be maintained at temperatures nearly 10 °C higher than the cellular environment (Chretien et al. 2017).

In addition, cells can purposely augment thermogenesis by expressing proteins promoting mitochondrial heat production. For example, uncoupling proteins can further increase proton leak, as illustrated by brown fat thermogenesis in mammals (Busiello et al. 2015). Or, a cell might express alternative oxidases to allow greater flux of electrons through the ETC without maximal capture of energy through proton pumping, resulting in the conversion of residual energy to heat (Moore and Siedow 1991). This approach facilitates thermogenesis by some flowering plants (Wagner et al. 2008) and can help maintain plant tissues at up to 35 °C above ambient temperature (Knutson 1974). Uncoupling proteins, like all proteins of the mitochondrial carrier family, are likely an eukaryotic invention (Haferkamp and Schmitz-Esser 2012). Alternative oxidases, however, are also encoded by prokaryotes (Pennisi et al. 2016), including by several α-proteobacteria (Roberts et al. 2004; Atteia et al. 2004).

## Heat Generation Provides an Immediate Selective Advantage for Proto-Mitochondrion Maintenance During Eukaryogenesis

I suggest a scenario in which a respiring proto-mitochondrial endosymbiont was encountered and completely enveloped by an archaeal host typically resident at high temperatures. Phylogenomic analyses imply that the archaeal host contributing to the formation of eukaryotes may have emerged from the recently discovered ‘Asgard’ superphylum of archaea (Zaremba-Niedzwiedzka et al. 2017), although the precise relationship between these organisms and eukaryotes requires further elaboration (Da Cunha et al. 2017). Most knowledge regarding the ‘Asgard’ superphylum has been obtained by the study of genomic fragments recovered from organisms within the Lokiarchaeota clade. Lokiarchaeal sequences have been recovered from sediments near a hydrothermal vent (Spang et al. 2015), and the ancestors of Lokiarchaeota and other Asgard members were thermophilic (Zaremba-Niedzwiedzka et al. 2017; Williams et al. 2017), consistent with the idea that a Lokiarchaeota-related organism might have been the host of the proto-mitochondrial endosymbiont. Lokiarchaeota express the ancient Wood–Ljungdahl pathway (Sousa et al. 2016; Williams et al. 2017), utilized by both autotrophic and heterotrophic organisms (Schuchmann and Müller 2016). Consequently, two generalized metabolic scenarios based upon endosymbiont occupation of a Lokiarchaeota-related host are plausible. An autotrophic host might have utilized H_2_ and CO_2_ to produce acetyl-CoA and downstream products for consumption and oxidation by the endosymbiont. Alternatively, both host and proto-mitochondrion may have fed upon organic carbon. Lokiarchaeota and other Asgard members have not yet been cultivated, and so the metabolic strategies used by these organisms are not yet fully revealed.

**Fig. 1:**
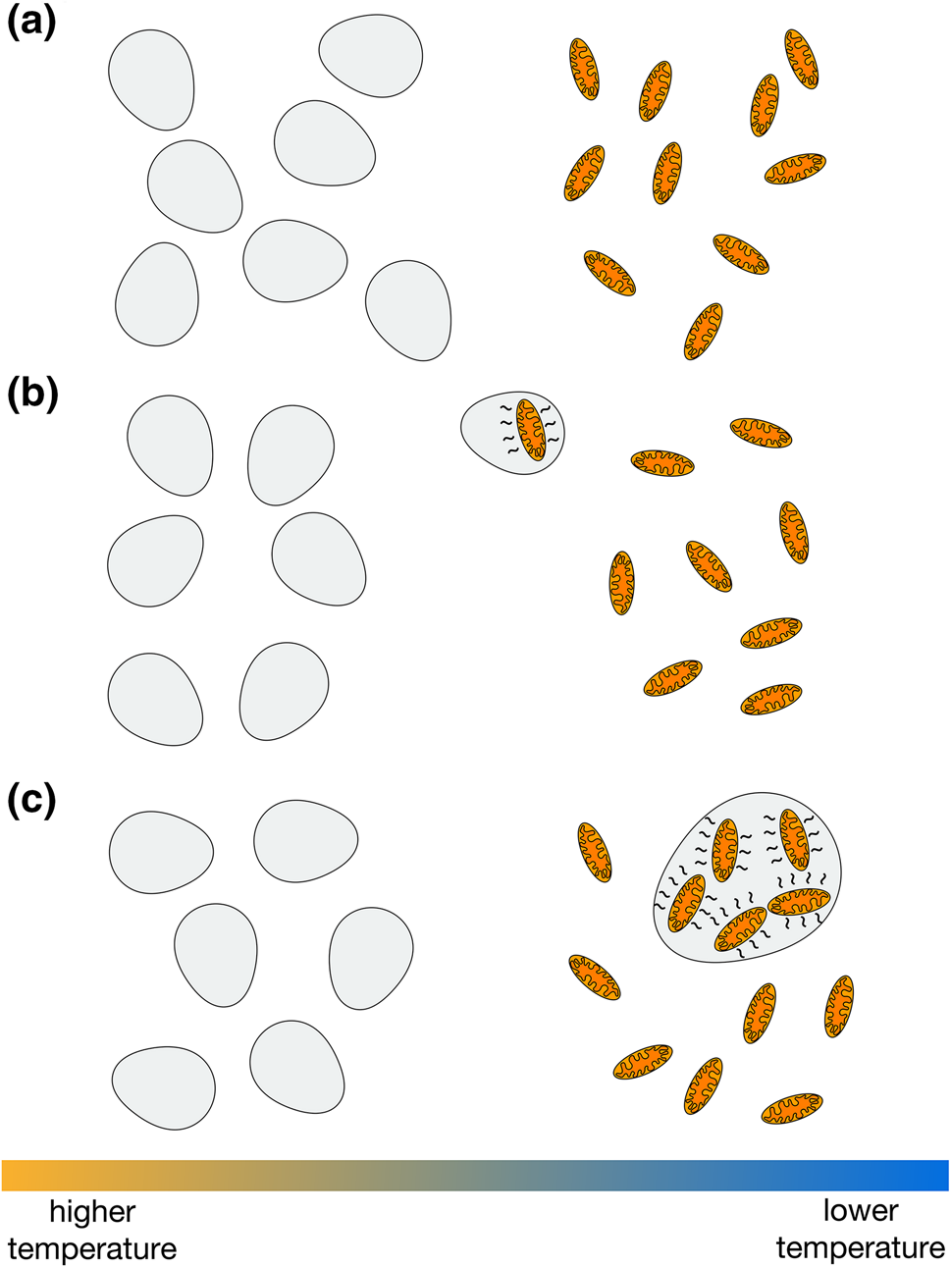
Internalization of heat-generating bacteria could permit archaeal colonization of cooler environments. **a** Ancestral archaeal cells eventually forming the proto-eukaryotic host (gray) would initially be limited to proliferation at higher temperatures. **b** The archaeon would encounter and enclose a respiring proto-mitochondrial endosymbiont (orange). **c** After sufficient endosymbiont load has been achieved, and the heat generated by electron transport and proton leak reaches a sufficient value, the proto-eukaryote may withstand lower temperatures

Immediately after entry, the proto-mitochondrion need not have provided any particular advantage to its host, and might have even been a parasite rather than an endosymbiont. However, upon colonization of a novel, cooler environment, the collection of heat-generating structures enclosed within the plasma membrane would allow the host to maintain the cell’s internal temperature at a value higher than ambient (Fig. 1). Heat would be generated by dissipation of energy during passage of electrons through the ETC, and indeed it has been suggested that the ETCs of endosymbionts and parasites may have increased latitude to ‘waste’ energy as heat (Schoepp-Cothenet et al. 2013). In addition, protons pumped to the bacterial periplasm by the ETC or by operating the ATP synthase in reverse (Dimroth and Cook 2004; Campanella et al. 2009) could leak through the bacterial IM, thereby intensifying heat production. Upon movement of the proto-eukaryote to a cooler location, this proposed scenario allows an immediate cooperative advantage for both host and endosymbiont. The host cell would receive heat required to endure or colonize a lower temperature niche, and the endosymbiont would obtain sufficient metabolites from the host to allow continued heat generation and to support its own maintenance. By contrast, although ATP synthesis is a prominent function of mitochondria, and a link between robust mitochondrial ATP production and eukaryotic complexity can be envisioned (Lane and Martin 2010; Martin et al. 2017), views of initial proto-mitochondrion establishment based on an exigent need for endosymbiont ATP production have been viewed with skepticism. First, one must propose that the host cell was incapable of fulfilling its ATP needs under selection and that the endosymbiont generated more ATP than it required before encountering the proto-eukaryotic host (Martin and Müller 1998). Second, one must assert that this endosymbiont was initially prepared and willing to export its ATP to the host, in spite of an initial lack of the antiporter currently used to exchange cytosolic ADP for ATP (Karlberg et al. 2000) and in the face of evidence suggesting that intracellular bacteria closely related to mitochondria may be unwilling to share ATP with host cells (Winkler and Neuhaus 1999).

## A Move Toward Complexity at Lower Temperatures

As this proposed partnership allowed movement of host cells and their resident endosymbionts to colder climates, the apparent barriers to genome size and diversity presented by life at high temperatures (Laksanalamai et al. 2004; Friedman et al. 2004; Drake 2009; Groussin and Gouy 2011; Sabath et al. 2013) would have been circumvented. Moreover, the arrangement I propose may have set the stage for further progress toward the cellular complexity characteristic of eukaryotes.

**Fig. 2:**
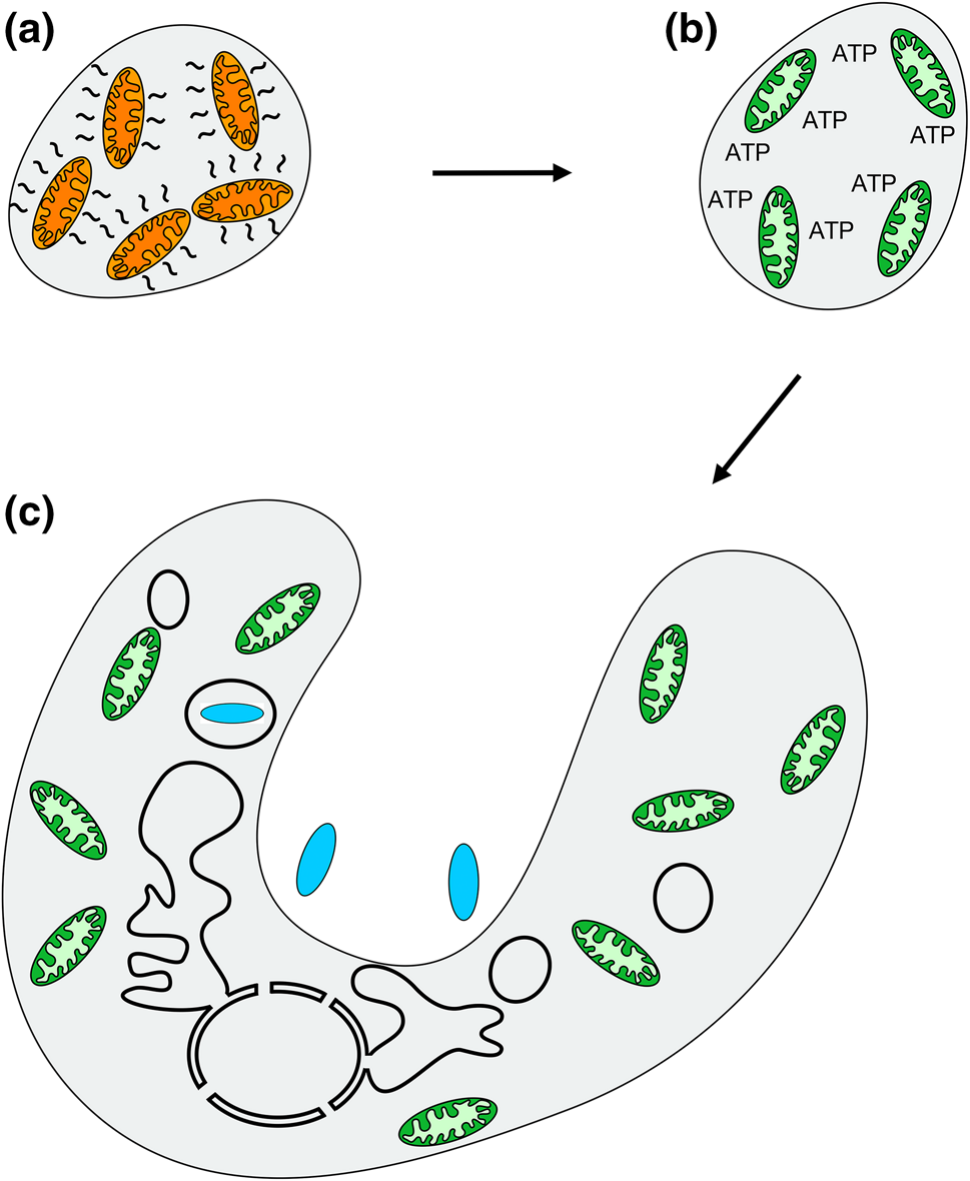
A subsequent switch to higher ATP generation capacity could promote increased cellular complexity. **a** After initially promoting heat generation and permitting movement of the proto-eukaryote to a cooler location, subsequent genetic changes obviate the need for maximal endosymbiont heat production. **b** Tighter coupling of electron transport to ATP synthesis then evolves, resulting in increased ATP abundance. **c** Higher ATP output from mitochondria leads to increased subcellular compartmentalization and promotes the ability to phagocytose other cells (prey prokaryotes in blue)

First, after the early eukaryote had initially colonized environments of lower temperature, further genetic changes and acquisitions would have rendered unnecessary a priority on proto-mitochondrial heat generation. Subsequently, better coupling of ETC activity to ATP synthesis, coincident with the introduction of an antiporter exchanging cytosolic ADP for ATP synthesized in the mitochondria, would have allowed greater ATP availability to the early eukaryotic cell (Fig. 2). While debate continues regarding the possibility that the archaeal host was capable of phagocytosis before encountering the proto-mitochondrial endosymbiont, higher ATP concentration may have promoted the ability to prey upon other cells already resident in the new niche of the proto-eukaryote. The acquired nutrients could then be directed toward maintenance of a more elaborate subcellular organization and increased cell mass, further promoting a predatory lifestyle for the proto-eukaryote (Stanier 1970; Martin et al. 2017). In addition, while a matter of contention (Lane and Martin 2010; Lynch and Marinov 2015, 2017; Lane 2017), increased ATP availability may have led to augmented protein synthesis capacity and to a corresponding expansion in gene content. Supporting these possibilities, oxygen solubility increases with reduced temperature (Ming and Zhenhao 2010), and therefore movement to a cooler environment could increase ATP production linked to oxidative phosphorylation while also allowing for a basal level of heat output.

**Fig. 3:**
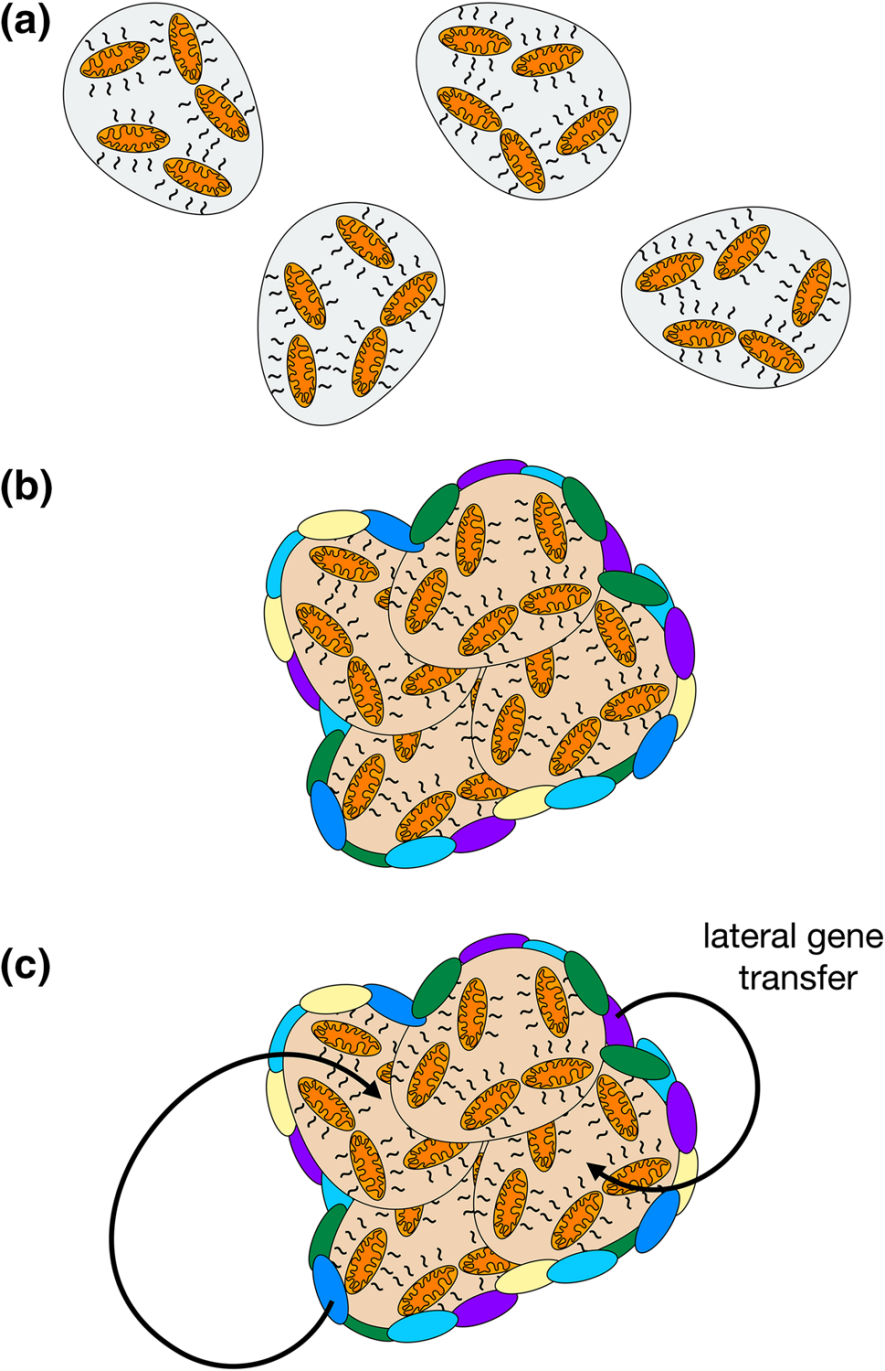
The need to avoid heat loss may indirectly encourage LGT from bacteria to the proto-eukaryote. **a** Single cells carrying heat-generating endosymbionts are thought to rapidly equilibrate their temperature with the environment. **b** However, archaea often form mixed aggregates that include bacteria (colored ovals), and archaea-containing biofilms can be of significant size (not reflected here). By decreasing the surface-area-to-volume ratio, a greater amount of endosymbiont-generated heat might be preserved by cells (reflected by red cytoplasm). **c** The formation of large conglomerates of cells would facilitate LGT to the early eukaryotic cell while encouraging heat retention

Second, it has been suggested that single cells are generally in temperature equilibrium with their environment (Johnson et al. 2009; Baffou et al. 2014), although arguments focused only upon heat flow from the cell, while ignoring the relative ease of raising and maintaining the temperature of a small cell volume, may not fully reflect the possibility of heating a single cell through metabolism. In any case, formation of extensive multicellular clusters with a reduced surface-area-to-volume ratio, if containing enough cells (Baffou et al. 2014), could certainly promote the retention of endosymbiont-generated heat. Indeed, large multicellular aggregates and biofilms, consisting of both archaea and bacteria, are commonplace (Fröls 2013), and large-scale LGT between members of a heat-conserving conglomerate may have contributed to the transfer of genes to the proto-eukaryote from prokaryotic sources beyond the proto-mitochondrion (Fig. 3) (Gabaldón and Huynen 2003; Kurland et al. 2006; Booth and Doolittle 2015; Gray 2015).

**Fig. 4:**
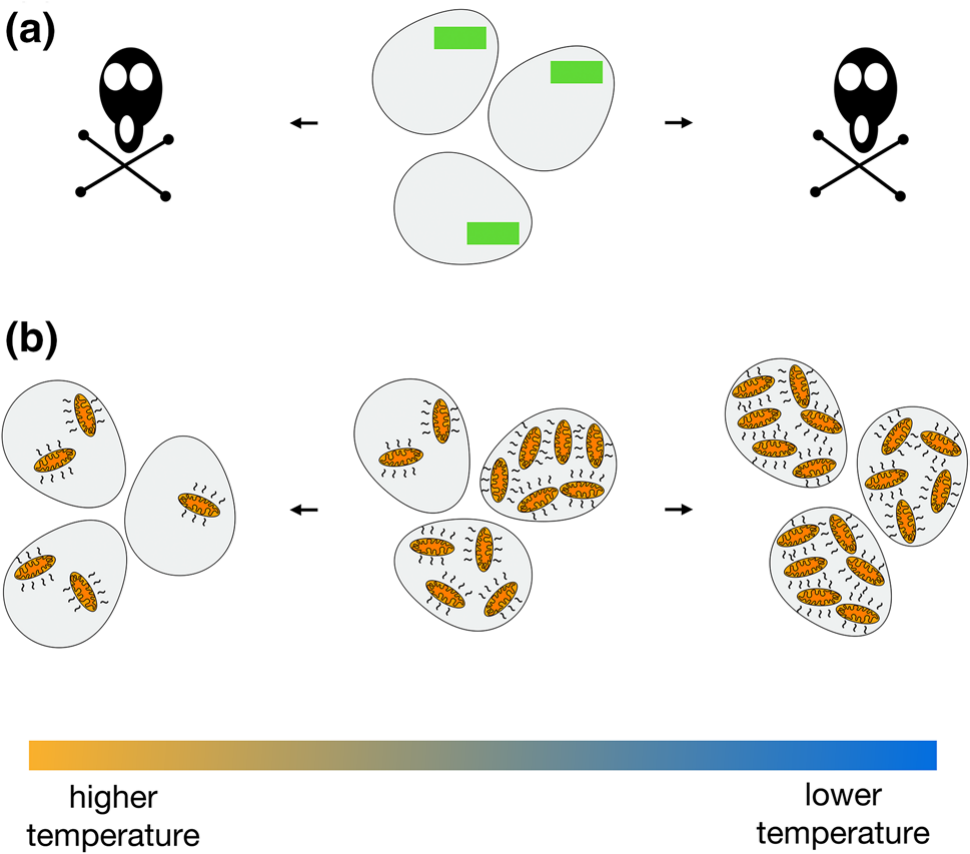
Cell-to-cell variability in the number of heat-producing endosymbionts may allow a population to be resilient to environmental temperature changes. **a** As an example, a population of cells adapted to narrow temperature range by a fixed genotype (green bar) may not be able to easily endure or colonize environments of significantly different temperature. **b** However, inherent variability in endosymbiont load among a population of proto-eukaryotes might allow at least some members of an otherwise genotypically identical population to subsist or thrive at more widely divergent temperatures

Finally, I suggest that proto-mitochondrial heat production provided additional flexibility to the eukaryotic ancestor population that would be difficult to obtain by fixation of mutations and gene transfers. Since one might expect stochastic differences in the quantity of heat-producing endosymbionts among a population of proto-eukaryotic cells, such a population might be resilient in the face of environmental temperature changes. Upon encountering lower temperatures, those cells with more heat-producing endosymbionts would flourish, and conversely, upon meeting higher temperatures, those cells with a more limited endosymbiont load would prosper (Fig. 4), thereby maintaining a continuous lineage of proto-eukaryotes. Additionally, genotypic heteroplasmy, with some endosymbiont ETCs better coupled to ATP synthesis than others, would allow further tailoring of heat production following selective pressure. Later, the cell might evolve mechanisms to control endosymbiont load in a bid to carefully balance heat generation with the environmental temperature. It is plausible that the need to curb the abundance of heat-producing endosymbionts was a driving force for the evolution of autophagy, since this process, like mitochondria, appears to have been characteristic of the last eukaryotic common ancestor (Yang et al. 2017).

## Conclusion

As highlighted in this work, mitochondria can be a significant source of heat production, and the ability to convert energy from electrons into heat may have been the earliest basis for integration of the proto-mitochondrion with its archaeal host. Such a scenario bridges a conceptual gap between endosymbiont entry and the eventual utility of mitochondrial ATP generation in fostering increased complexity. Strong support for this model would be obtained by discovery of a modern-day intracellular endosymbiont that currently provides heat to its host organism. In addition, phylogenetic analyses comparing genes and proteins from mitochondria, bacterial relatives of mitochondria, and the archaeal kin of eukaryotes, with a focus on temperature-related parameters such as guanine-cytosine content, amino acid usage, and activity of reconstructed ancestral proteins (Akanuma 2017), can provide insight regarding the proliferation temperatures of our closest archaeal and bacterial ancestors. Finally, continued investigation of subcellular heat generation and distribution at the experimental and theoretical levels will be instructive regarding a potential role for endosymbiont heat production during eukaryogenesis.

Beyond their roles in bioenergetics, mitochondria are the location of other widely conserved cellular processes. For example, iron–sulfur cluster generation is a primary function of mitochondria (Karnkowska et al. 2016; Braymer and Lill 2017), and reactions important for lipid metabolism or amino acid production can also be compartmentalized at these organelles (Makiuchi and Nozaki 2014; Ahn and Metallo 2015; Tatsuta and Langer 2017). While the mitochondrion’s role in stripping energy from electrons was undoubtedly significant during the emergence of eukaryotes, a broader focus on the many functions of mitochondria lying outside of the respiratory chain will be informative when considering early eukaryotic evolution.
